# Hybrid gold-iron oxide nanoparticles as a multifunctional platform for biomedical application

**DOI:** 10.1186/1477-3155-10-27

**Published:** 2012-06-25

**Authors:** Clare Hoskins, Yue Min, Mariana Gueorguieva, Craig McDougall, Alexander Volovick, Paul Prentice, Zhigang Wang, Andreas Melzer, Alfred Cuschieri, Lijun Wang

**Affiliations:** 1 Institute for Medical Science and Technology (IMSaT), University of Dundee, Wilson House, 1 Wurzburg Loan, Dundee, DD2 1FD, UK; 2 Division of Electronic Engineering and Physics, University of Dundee, Nethergate, Dundee, DD1 4HN, UK

**Keywords:** Magnetic nanoparticles, Gold nano-shells, Magnetic resonance imaging, Surface plasmon resonance, Multifunctional nanoparticles

## Abstract

**Background:**

Iron oxide nanoparticles (IONPs) have increasing applications in biomedicine, however fears over long term stability of polymer coated particles have arisen. Gold coating IONPs results in particles of increased stability and robustness. The unique properties of both the iron oxide (magnetic) and gold (surface plasmon resonance) result in a multimodal platform for use as MRI contrast agents and as a nano-heater.

**Results:**

Here we synthesize IONPs of core diameter 30 nm and gold coat using the seeding method with a poly(ethylenimine) intermediate layer. The final particles were coated in poly(ethylene glycol) to ensure biocompatibility and increase retention times *in vivo*. The particle coating was monitored using FTIR, PCS, UV–vis absorption, TEM, and EDX. The particles appeared to have little cytotoxic effect when incubated with A375M cells. The resultant hybrid nanoparticles (HNPs) possessed a maximal absorbance at 600 nm. After laser irradiation in agar phantom a ΔT of 32°C was achieved after only 90 s exposure (50 μgmL^-1^). The HNPs appeared to decrease T_2_ values in line with previously clinically used MRI contrast agent Feridex®.

**Conclusions:**

The data highlights the potential of these HNPs as dual function MRI contrast agents and nano-heaters for therapies such as cellular hyperthermia or thermo-responsive drug delivery.

## Background

Iron oxide (magnetite, Fe_3_O_4_) nanoparticles (IONPs) have been the focus of extensive investigation over the past decade [1-3]. Fe_3_O_4_ possesses inherent magnetic properties which are desirable for a large range of biomedical applications including cellular sorting [4], targeted drug delivery [5], tissue engineering [6] and most commonly as magnetic resonance imaging (MRI) contrast agents [7-10]. When used as a contrast agent the high saturation magnetism of the IONPs results in increased transverse relaxivity [11,12]. The increased transverse relaxivity (decreased T_2_ signal) is observed as a darker area in an MRI scan [13]. Although a range of iron oxide nanoparticles such as Feridex® [14] and Resovist® were previously clinically approved [15], on-going concern was placed on the long term toxicity of these particles *in vivo*[16]. Hence, recently both Feridex® and Resovist® have been withdrawn from use in humans. Degradation of iron oxide into iron ions in physiological environments [17] has been reported to increase free radical production in cells causing damage which may lead to cell death [18-20]. Commonly IONPs are coated with organic macromolecules such as poly(acrylic acid) (PAA) [21], dextran [22] and poly(ethylenimine) (PEI) [23] or inorganic coatings such as silica [24], carbon [25] or precious metals (e.g. gold or silver) [26].

Gold is both chemically stable and biocompatible [27]. Gold nanoparticles (AuNPs) have been widely acknowledged to possess unique optical properties [28]. When nanoparticles of 10–100 nm in size are exposed to optical radiation, the electrons within the AuNPs resonate causing them to absorb and scatter light [29]. This phenomenon is known as surface plasmon resonance (SPR) where the optimal resonance wavelength is determined by the nanoparticle construct (size, shape, surface area, surface coating etc.) [28,29]. When nanoparticles are irradiated, the SPR absorption of Au nanoparticles is followed by rapid conversion of light into heat [28]. In biomedicine this unique property can be exploited for applications such as photo-thermal ablation [29] and thermo-sensitive drug delivery [29]. Clinically, the optimal wavelengths for laser irradiation of AuNPs are within the ‘biological near infrared region (NIR, 750–1400 nm)’ [30]. Laser radiation within the NIR window are capable of deep tissue penetration due to the high transmission of water and haemoglobin within these wavelengths [30], this can be exploited for non-invasive or minimally invasive therapy. Other gold nanostructures such as nano-shells and nano-spheres have been reported [28], here the hollow architecture improves SPR resulting in a stronger absorption in the NIR region [31]. The increased absorption of gold nano-shells is a result of the interactions between the plasmon, supported by both the inner and outer surfaces of the shell [32,33]. The physical properties of gold nano-shells are highly tuneable depending on shell thickness and functionalization [34]. Increased shell thickness has been shown to result in decreased absorption and hence decreased heating [28,34].

When gold is used to coat IONPs the outer gold shell acts as a barrier preventing core oxidation and enzymatic degradation [35]. Hybridization of IONPs with gold results in a multimodal platform which benefits from the unique properties of both materials [30,35]. The gold exterior shell also provides an anchorage site for further functionalization [30]. The ability to achieve accurate real-time imaging complimentary to localized tissue heating is highly advantageous for applications such as tumour ablation and drug delivery [30,36]. Direct coating of gold nano-shells onto IONPs is often problematic due to lack of control over physical characteristics such as shell thickness and geometry [35]. Direct coating has also been reported to decrease the saturation magnetisation, hence reducing the relaxivity of the IONP core and reducing its ability to act as a MRI contrast agent [12]. These mechanisms are not well understood but may be due to migration of gold atoms into the magnetic IONP core [12]. Furthermore, increased shell thickness has an unfavourable effect on longitudinal and transverse relaxivities [12].

Recently Goon and colleagues reported the fabrication of iron oxide-gold core-shell hybrid nanoparticles (HNPs) using a polymer intermediate separating the two entities [27]. Addition of an organic intermediate layer between the IONP and gold shell is expected to prevent gold migration into the core, increasing the saturation magnetisation and relaxivity [12]. Gold coating of the polymer coated IONPs is achieved by firstly attaching gold seeds to the nanoparticle surface followed by subsequent reduction of gold onto the surface forming a complete coat [27,37]. The seeding method allows for a greater degree of control over shell thickness [27,37].

Here we report the synthesis and physicochemical properties of Fe_3_O_4_-PEI-Au-PEG (HNPs). The IONP core is firstly coated with poly(ethylenimine) (PEI, MW 750,000) which acts as an intermediate between the core and shell. Gold coating is achieved via the seeding method. A thiol (−SH) capped poly(ethylene glycol) (PEG) is finally used to functionalize the gold surface. PEG is an FDA approved polymer which is known to disguise nanoparticles, masking them from the immune system hence increasing blood circulation time [38]. Both chemical and biological characterization will be carried out to determine the suitability of these particles for biomedical use. Finally, the potential of these particles to act as multimodal platforms for use as ‘nano-heaters’ and MRI contrast agents will be demonstrated using laser irradiation and magnetic resonance using a 1.5 T clinical MRI.

## Results

### Synthesis and characterization of HNPs

**Figure 1:**
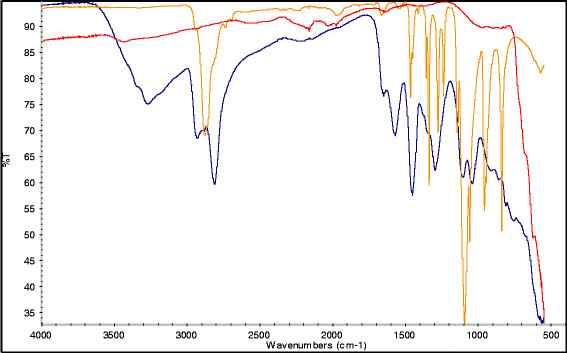
**FTIR spectra of A) Fe**_**3**_**O**_**4**_**(red), B) FE**_**3**_**O**_**4**_**-PEI (blue) and C) Fe**_**3**_**O**_**4**_**-PEI-Au-PEG (orange).** Analysis carried out on Nicolett iS5 FTIR with diamond tip iD5 ATR attachment (Thermo Scientific, UK). Samples were freeze dried prior to analysis and 64 scans were run.

The Fe_3_O_4_ particles were successfully synthesized and coated with PEI, gold and subsequently PEG. Inductively coupled plasma – optical emission spectroscopy (ICP-OES) was used to deduce the concentration based on the total iron and gold content of the nanoparticles (NPs) (Table 1). Fourier transform infrared spectroscopy (FTIR) analysis of the freeze dried particles indicates that PEI attachment to the iron oxide surface had occurred. The PEI gave rise to –NH peaks at 3300, 1700 & 1600 cm^-1^ and a distinct C-N peak at 1000 cm^-1^ (Figure 1). The broad peak observed at 3100 cm^-1^ was due to free water which was still present in this hygroscopic polymer even after 8 h freeze drying. After PEGylation of the gold coated polymer, a peak was observed at 2800 cm^-1^ which indicated the presence of the PEG moiety due to the alkyl chain of the polymer backbone. Additionally a small peak was observed at 3400 cm^-1^ which was due to the C = O stretch of the bonds in the PEG moiety, these findings suggested the surface functionalization of the HNPs was successful.

**Table 1:** Physicochemical properties of reaction steps in HNP formation using photon correlation spectroscopy (PCS), zeta potential measurement and inductively coupled plasma-optical emission spectroscopy (ICP-OES)

**Particle**	**Hydrodynamic Radius nm ± SD**	**Polydispersity Index ± SD**	**Zeta Potential mV ± SD**	**Concentration determined by Inductively Coupled Plasma – Optical Emission Spectroscopy mgmL**^**-1**^**(Average value, n = 2)**	
				**Fe**	**Au**
Fe_3_O_4_	1112 (97)	0.763 (0.104)	-16.9 (0.379)	14.5	-
Fe_3_O_4_-PEI	237 (2)	0.194 (0.030)	+55.6 (0.702)	13.5	-
Fe_3_O_4_-PEI-Au_seed_	309 (12)	0.363 (0.016)	+35.0 (0.115)	1.86	3.36
Fe_3_O_4_-PEI-Au_coat_	132 (37)	0.792 (0.156)	-23.3 (1.950)	2.74	3.53
Fe_3_O_4_-PEI-Au-PEG	139 (71)	0.332 (0.072)	-21.7 (0.551)	2.20	3.06

**Figure 2:**
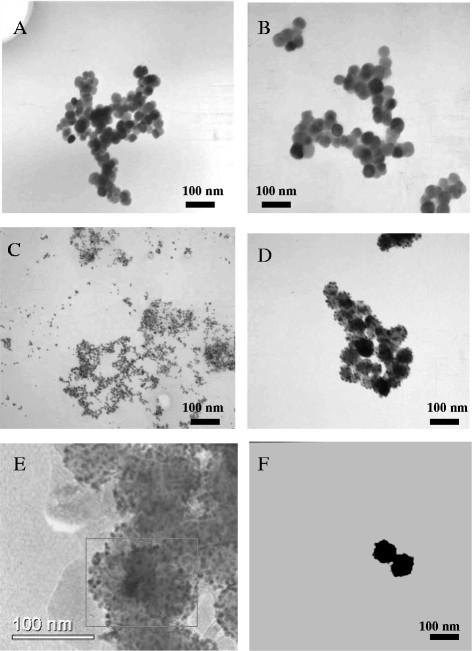
**TEM images of A) Fe**_**3**_**O**_**4**_**B) Fe**_**3**_**O**_**4**_**-PEI, C) Gold seeds, D) Fe**_**3**_**O**_**4**_**-PEI-Au**_**seed**_**, E) Fe**_**3**_**O**_**4**_**-PEI-Au**_**seed**_**(HR-TEM) and F) Fe**_**3**_**O**_**4**_**-PEI-Au**_**coat**_.

**Figure 3:**
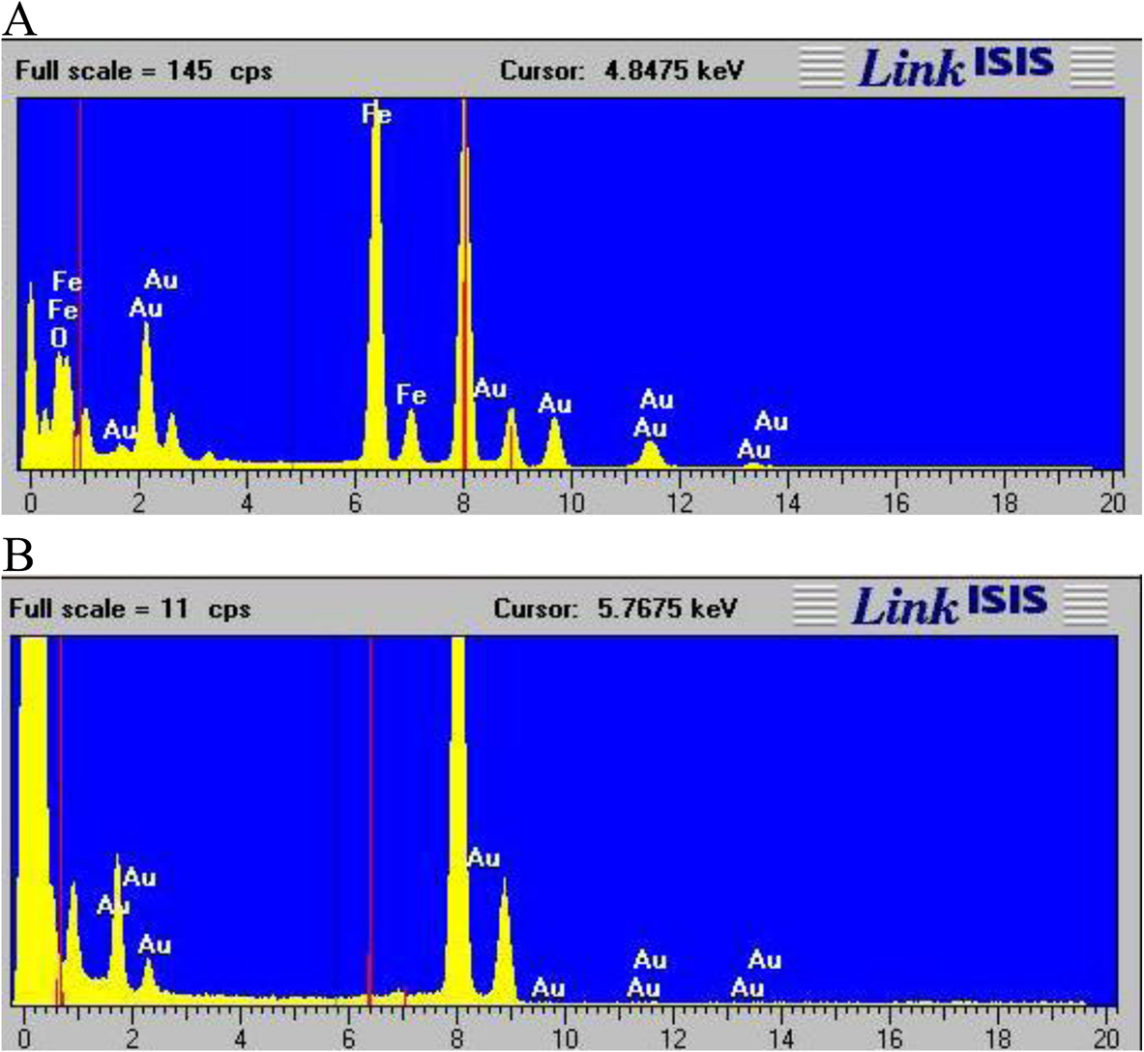
**EDX analysis of particle constituents of A) Fe**_**3**_**O**_**4**_**-PEI-Au**_**seeds**_**and B) Fe**_**3**_**O**_**4**_**-PEI-Au**_**coat**_**.** Analysis carried out using a Jeol JEM 2010 with EDX ISIS analysis software.

The ‘naked’ Fe_3_O_4_ had a hydrodynamic radius of 1112 nm determined by photon correlation spectroscopy (Table 1). This large value indicated that large aggregates had formed in solution due to the inherent magnetic properties of the IONPs. The TEM micrograph gave a more realistic representation of the Fe_3_O_4_ size which was approximately 30 nm (Figure 2A). PEI coating of the nanoparticles reduced the hydrodynamic radius to 237 nm, this indicated an increase in solution stability, and the significant decrease in polydispersity index from 0.763 to 0.194 confirmed this assumption (Table 1). The TEM images of the Fe_3_O_4_-PEI showed a slight increase in particle diameter to approximately 40 nm (Figure 2B). Gold seeds were synthesised (Figure 2C) and attached onto the Fe_3_O_4_-PEI surface by electrostatic interactions, the resultant particles can be seen in Figure 2D & E. The gold seeds gave rise to a unique ‘bobbly’ surface. EDX and ICP analysis of the Fe_3_O_4_-PEI-Au_seed_ showed the presence of both Au and Fe (Figure 3A & Table 1), the latter of which was not observed in the EDX spectra of the completely coated NP (Figure 3B). The gold coated HNPs can be seen in Figure 2F, these NPs possessed a large polydispersity index of 0.792, and this value indicated that there were a large number of size populations in solution (Table 1). This phenomenon is perhaps due to gold agglomeration [39]. After surface functionalization with PEG the PDI was reduced to 0.332 and the hydrodynamic radius of the final particle was 139 nm (Table 1). We estimated by examining the TEM images that the thickness of the gold coating was approximately 10–15 nm.

The Fe_3_O_4_ possessed a negative surface charge of −16.9 mV determined by zeta potential measurement. This negative value can be attributed to surface sulphate associations from the synthesis precursors [20] (Table 1). After PEI coating of the Fe_3_O_4_ the zeta potential increased to +55.6 mV due to the positive amine groups of the polymer backbone; this large increase gives good indication that coating was successful. The gold seeded and fully coated NPs experienced a reduction in surface potential caused by the negatively charged gold atoms (+35.0 and −23.3 mV respectively) this was further reduced to −21.7 mV after surface coating with PEG (Table 1). The decrease in zeta potential after PEGylation can be attributed to the presence of –OH groups on the PEG coating as previously reported [19,20].

**Figure 4:**
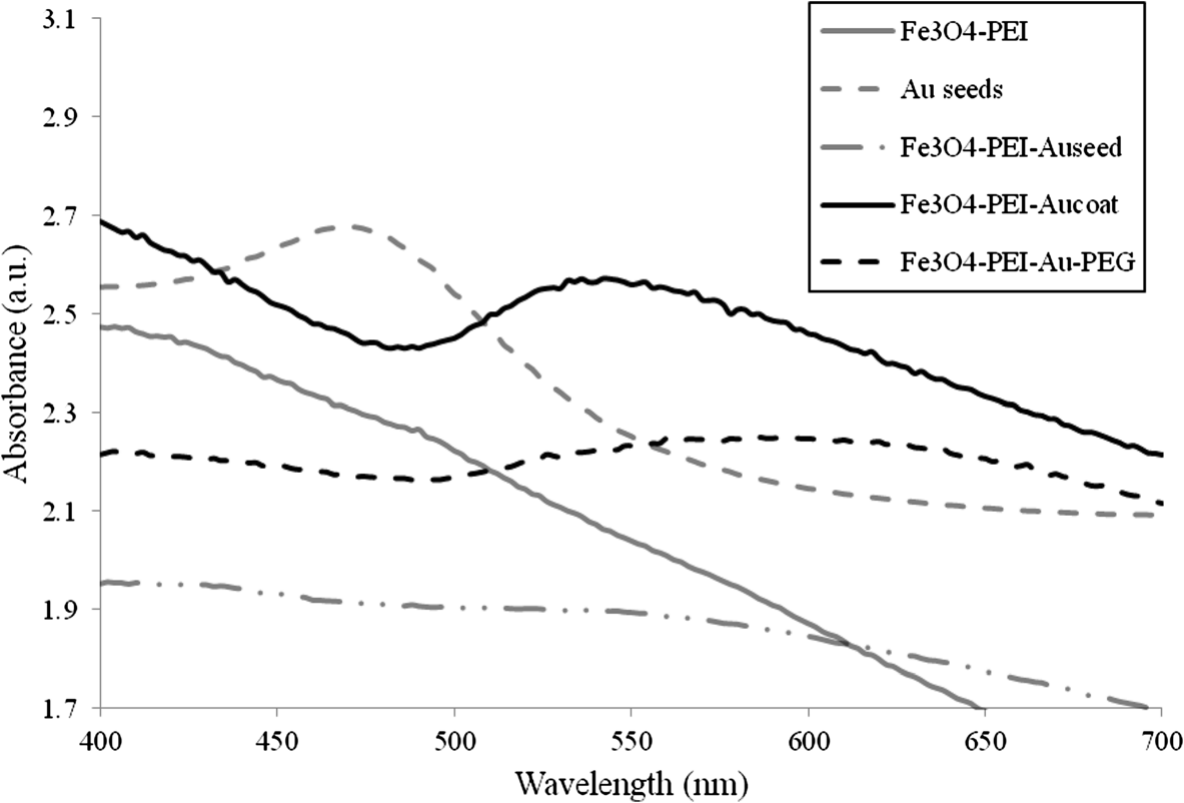
**Representative UV–vis absorbance spectra (arbitrary units, a.u.) of nanoparticles of Fe**_**3**_**O**_**4**_**-PEI, Au seeds, Fe**_**3**_**O**_**4**_**-PEI-Au**_**seed**_**, Fe**_**3**_**O**_**4**_**-PEI-Au**_**coat**_**and Fe**_**3**_**O**_**4**_**-PEI-Au-PEG.**

Au NPs can absorb light and convert it into localized heat for potential photo-thermal applications. UV-visible spectroscopy was used to obtain absorption spectra of the particles (Figure 4) in order to determine the wavelength at which maximum absorbance occurred (λ_max_). The λ_max_ value is used as an indication of the wavelength at which the surface plasmon resonance effect will be optimal. The initial scan of PEI-coated iron oxide cores produced featureless spectra indicating an absence of surface plasmon resonance (SPR).The gold seed solution possessed a λ_max_ of 480 nm, this was red shifted upon conjugation to the Fe_3_O_4_-PEI surface to 520 nm (however this was difficult to observe at the concentration tested, 1 mgmL^-1^). After complete coating had been achieved a small shift to 540 nm was observed, a further red shift was observed after the final PEG coating had been attached with a final λ_max_ of 600 nm exhibited. The red shifting occurring during each step of the synthesis is further indication that the fabrication of HNPs had been successful.

### Biocompatibility of HNPs

**Figure 5:**
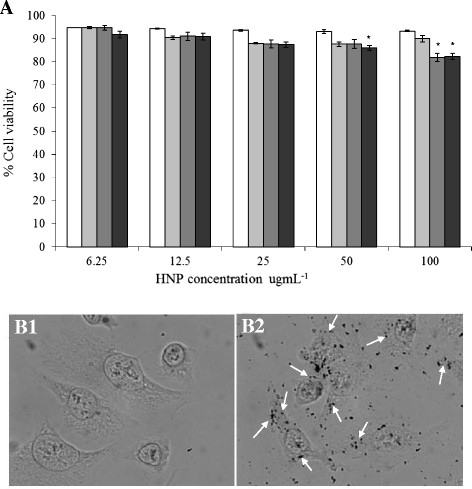
**Cellular interactions of HNPs with A375M cells.A**) Cell viability estimated by Trypan blue exclusion assay. Cells incubated with particles (6.25 – 100 μgmL^-1^) for □ 24, ■72, ■120 and ■168 h (n = 3, ±SE). Values of viability of treated cells were expressed as a percentage of that from corresponding control cells. **B**) Cellular uptake of in A375M cells. Particles (50 μgmL^-1^) incubated for 24 h before fixation and silver enhanced staining. Arrows indicate nanoparticles that bound to the cells (on cell membrane or inside the cells). * Denotes significant values compared to control (p < 0.05).

Cell viability of A375M cells incubated with HNPs was determined by Trypan blue exclusion assay (Figure 5A3). In general, the particles exhibited a dose and time responsive effect on cell viability. No significant reduction in viability was observed over the 7 day period up to 25 μgmL^-1^ (p > 0.05) and the HNPs did not exhibit apparent toxicity to those cells after 5 day incubation at concentrations up to 50 μgmL^-1^ (p > 0.05). After 5 day incubation with the highest concentration of particles (100 ugmL^-1^) an 18-20% decrease in viability was observed (p<0.05). These results indicate that the HNPs did not possess a highly toxic nature. Cellular uptake of HNPs in A375M cells was imaged by silver enhanced staining. The silver reagents become nucleated upon contact with the gold coating of the HNPs that were degraded by acid treatment resulting in precipitation of metallic silver viewed as a dark brown-to-black signal under the bright field light microscope. Figure 5B1 shows fixed A375M control cells. No dark spots due to nanoparticle presence was evident. Figure 5B2 shows cells incubated with 50 μgmL^-1^ for 24 h (95% viability, Figure 5A). A large number of dark dots over the image indicate nanoparticle presence. These particles appear to be mostly inside the cell or on the cell surface, as represented by those indicated by the arrows. Some particles appear in the surrounding areas this may be due to the adhesive nature of polymer-coated nanoparticles. Although we washed the cell cultures thoroughly before fixation and further processing we still could not achieve a complete elimination of nanoparticles that attached to the cell culture surface. We appreciate that this is a problem that many studies have encountered when dealing with cell incubation with polymer-coated nanoparticles. When compared with the control cells, the cells incubated with HNPs appear to be of similar physiology indicating that no morphological changes occurred upon nanoparticle exposure.

### Plasmonic hyperthermia of HNPs in response to laser treatment

In order to investigate the potential of the HNPs as nano-heaters, suspensions of HNPs in agar were irradiated with 532 nm light emitted by a continuous wave laser. This wavelength was chosen as it was relatively close to the λ_max_ value of the PEG coated HNPs (600 nm) determined by UV spectroscopy (Figure 4). The agar phantom was used to mimic physiological conditions. We observed a localized heating of the sample in the region exposed to laser radiation by thermocouple measurement. The heating in samples with no HNPs present was negligible.

To test the localization of the laser induced heating, a second thermocouple was fixed on the edge of the phantom sample 14 mm from the laser focal point. No temperature increase (or decrease) was recorded from this thermocouple during laser irradiation (data not shown). This indicated that heating only occurred in the region exposed to the laser radiation. Indeed translation of the sample across the laser beam qualitatively indicated the same result (data not shown). The ability to deliver a localized treatment is desirable so that surrounding healthy tissues do not experience secondary heating resulting in unwanted damage [28].

**Figure 6:**
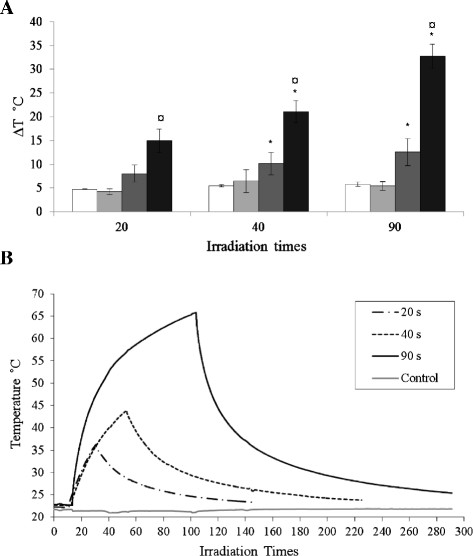
**Effect of laser irradiation on HNPs suspended in 2% agar gels.A**) Temperature change in particles (□ 0.4,■ 2, ■ 10 and ■ 50 μgmL^-1^) exposed to continuous green laser fixed at 532 nm (1.11 Watts, 7 mm beam focus) for 20, 40 and 90 s. ΔT was calculated as expressed in the Equation 1 (n = 3 ± SE). **B**) Representative real time temperature response curves of 50 μgmL^-1^ particles irradiated for 20, 40 and 90s. * Denotes significant difference compared with 20 s irradiation data and ¤ denotes significant difference of data of HNP concentration at 50 μgmL^-1^ compared with data of other concentrations of similar laser exposure (p < 0.05).

Figure 6A shows the change in temperature (ΔT) of the agar gels containing the HNPs (0.4, 2, 10, 50 μgmL^-1^), ΔT takes into account any temperature change in the control sample (Equation 1 in the Methods). The gels were heated for 20, 40 and 90 s. At concentrations of 0.4-2 μgmL^-1^, ΔT was approximately 5 °C after 20 s laser irradiation; upon longer laser exposure a small incremental time dependant increase was observed however, this was not significant (p > 0.05) . At 10 μgmL^-1^ & 50 μgmL^-1^ the ΔT significantly increased upon longer laser exposure (40 s & 90 s) when compared to 20 s irradiation. At 50 μgmL^-1^ the ΔT increased significantly at all exposure times compared to the ΔT data obtained for other concentrations (p < 0.05). Hence, the ΔT_max_ (31 °C) was observed at the highest concentration (50 μgmL^-1^) and longest exposure duration (90 s).

Figure 6B shows a representation of the real time temperature response pattern of the 50 μgmL^-1^ sample over the various irradiation times. The data further illustrates the time responsive nature of the heating effect. After laser irradiation the sample exposed to the laser for 20 s took almost 2 min to return to room temperature, after 40 s irradiation the sample took approximately 3 min (Figure 6B). After 4 min the particles heated for 90 s had not returned to their original temperature. These data thus provide important information for the choice of nanoparticle concentration and irradiation times for further studies where toxicity and ΔT would be the key elements.

### T_1_ & T_2_ MR relaxivity determined by magnetic resonance imaging (MRI) of HNPs

**Figure 7:**
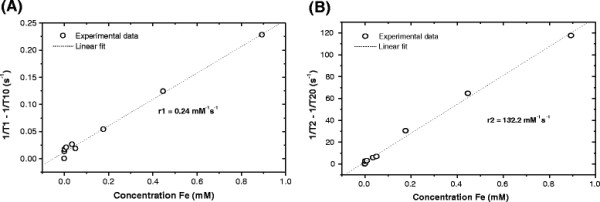
**Longitudinal (A) and transverse (B) relaxation rates as a function of concentration of Fe in HNPs.** The dotted line represents the linear fit to the experimental data. The relaxivities r1 and r2 were calculated as the gradient to the straight lines fitted to the experimental data.

Figure 7 shows the longitudinal (A) and transverse (B) relaxation rates (1/T_i_) as a function of concentration of Fe in mM. The corresponding longitudinal and transverse relaxivities r_1_ and r_2_ were calculated from the gradient of the straight lines fitted to those data. In both graphs the relaxation rates (1/T_i_) for the sample with the highest concentration of Fe (1.79 mM) had to be omitted as it deviated largely from the linear fit to the rest of the data. This deviation might be explained either with agglomeration of nanoparticles at such high concentrations or with the larger error introduced in the calculation of the T_2_ for that particular sample due to the much lower signal-to-noise ratio of the MR images obtained at longer TE.

This procedure of determination of r_1_ and r_2_ renders a transverse relaxivity of 132.2 mM^-1^ s^-1^ (Figure 7B). This value was similar to the relaxivity of previously clinically used Feridex®. Feridex® is a superparamagnetic nanoparticle coated with dextran; the transverse relaxivity of Feridex® is reportedly 133 mM^-1^ s^-1^ at 1.5 T magnetic field strength [40]. This data suggests that the gold coating on the surface of the IONPs does not impede their ability to act as magnetic resonance contrast agents. The relaxivity r_1_ of the HNPs was calculated to be 0.243 mM^-1^ s^-1^ (Figure 7A). This notably small r_1_ resulted in r_1_/r_2_ ratio of 0.0018. The reason for the small r_1_ value is not fully understood; perhaps the architecture of the HNPs may contribute to this phenomenon.

## Discussion

In this study we successfully synthesized iron oxide-gold hybrid core-shell nanoparticles. The iron oxide cores appeared to be monodisperse and around 30 nm in size (Figure 2A). Polymer coating was achieved using poly(ethylenimine) confirmed with FTIR and zeta potential measurement (Figure 1 & Table 1). Gold seeding was achieved using 2 nm gold nanoparticles and subsequently iterative gold reduction onto the nanoparticle surface resulted in formation of a full gold shell. Each step in the reaction was monitored with TEM (Figure 2), EDX analysis (Figure 3) and zeta potential measurement (Table 1). A gold coating of 10–15 nm thickness was achieved. However, this could be tailored in future to increase or decrease the thickness with further investigation of optimal physical properties for different purposes [28,34]. Finally, the hybrid particles were coated with PEG and had a hydrodynamic radius of 139 nm determined using PCS (Table 1). The red shifted UV–vis absorbance spectra also confirmed complete gold coating with final λ_max_ of 600 nm (Figure 4). Previous studies on gold nano-shells have reported that the λ_max_ can be red shifted into the infrared region 800–1200 nm [29]. Our particles did not appear to achieve such large shift. The absorbance peak obtained was very broad and spanned from 500–700 nm. The broad absorbance peak and the lack of shift up to the infrared region are perhaps due to the synthesis route, the presence of organic polymer layer between the gold coat and magnetic core and the variations in shell thickness or HNP diameter. Our results were however, comparable with others that a λ_max_ between 500–600 nm was achieved [12,27,35,36]. Ongoing work is being carried out in our lab to optimize particle parameters and external coatings in order to achieve narrower absorbance and further red shifted HNPs.

Cell viability assays indicated that the HNPs did not possess high cytotoxic effect on A375M cells over 7 day exposure (Figure 5). When compared to the cell viability data of polymer coated iron oxide [20] this was comparable to our previous study where we showed that after 7 days incubation with 100 μgmL^-1^ MNP-PEI-PEG only resulted in a 20% reduction in viability [20] (cell viability determined on three cell lines with similar observations). This finding indicates that the presence of the gold coating, or increased size particle diameter had little impact on the cytotoxic profile of these particles. Reports have suggested that gold coating protects the iron oxide core from enzymatic degradation which may result in free radical production [35]. Our findings provide increased confidence for the long term *in vivo* effects of these HNPs. Further stability studies and more *in vitro* assays are currently under way in our lab.

Few studies have demonstrated the heating effect of iron oxide-gold HNPs. Hirsh et al. previously developed core-shell iron oxide-gold hybrid particles [41]. The particles were administered into tumour tissue in mice and exposed to laser irradiation (820 nm, 4 Wcm^-2^, 5 mm spot diameter, < 6 min). Their experiments showed that irradiation of nanoshell-loaded tumours resulted in an average temperature increase of 37.4 °C after 4–6 min exposure. Hirsh reported that this temperature increase was above the threshold temperature at which irreversible tissue damage occurs [41]. Upon laser irradiation at 532 nm our HNPs showed a time and concentration dependant heating effect (Figure 6). This wavelength was outside the λ_max_ determined by UV spectroscopy (600 nm), however as previously mentioned the broad absorbance peak of the HNPs allowed that at 532 nm a degree of absorbance and scattering was still achieved. The data obtained hence demonstrate the potential of the HNPs for future use as nano-heaters. To determine the optimal heating effects for these particular particles further irradiation at 600 nm is on-going. We found that at the highest concentration (50 ugmL^-1^) and laser exposure time (90 s) the HNPs absorbed the light resulting in a ΔT of 32 °C. This increase was significantly larger than at the other concentrations tested (0.4, 2 & 10 ugmL^-1^) (p < 0.005). Our data was obtained in nanoparticles suspended in an agar phantom, whilst this was an *in vitro* assay, the agar was used to mimic *in vivo* tissue conditions. We appreciate that for *in vivo* applications, nanoparticle concentrations of 5–10 μgmL^-1^ would be much more realistic than 50 μgmL^-1^[42]. The heating effect of HNPs with particle concentrations at 5–10 μgmL^-1^ is now under more in-depth investigation.

Our results showed that our ‘custom made’ iron oxide coated with gold possessed a r_2_ (132 mM^-1^ s^-1^) comparable with previously clinically administered Feridex® (133 mM^-1^ s^-1^) [40]. Our data also suggests that the HNPs also resulted in a T_1_ reduction resulting in r_1_/r_2_ ratio of 0.0018. Previously, it has been suggested that gold coating of IONPs results in quenched contrast ability [12]. These results suggest that the magnetic properties of our HNPs after gold coating are in line with clinical standards and thus have a potential use as contrast agents. These findings coupled with the laser irradiation data highlight that the unique physical properties of the magnetic IONP and gold nano-shell (SPR) are preserved using a polymer ‘cushion’ layer between the core and shell. These findings along with the cell viability data indicate that our HNPs have clinical potential as multimodal platforms for a range of biomedical applications such as image guided cellular hyperthermia or thermo responsive drug delivery. On-going work in our lab is currently being carried out in order to further exploit the properties of these HNPs.

## Conclusions

This study demonstrates the potential of HNPs composed of an iron oxide core, poly(ethylenimine) intermediate and gold coating in controlled, highly localized tissue heating and as MRI contrast agent. The data indicate that the presence of the polymer spacer does not hinder the ability of the HNPs to act as multimodal platforms for use as nano-heaters and in MR imaging. More work is needed to further tune and exploit the potential of these hybrid structures in terms of the thickness of the gold shell, the cellular uptake and hyperthermia and the manipulation of laser treatment to obtain the optimal effects.

## Methods

### Synthesis of HNPs

Nitrogen was bubbled through a solution of sodium hydroxide and potassium nitrate dissolved in deionised water at 90 °C for 1 h. Iron sulphate dissolved in sulphuric acid (0.01 M) was added to the reaction and the mixture was stirred for 24 h at 90 °C under nitrogen. After this time the reaction was rapidly cooled on ice and the particles were washed 6 times with deionised water and magnetically separated from solution. The resultant particles (500 mg) were stored at 4 °C. Fe_3_O_4_ solution (5 mL) was added to 50 mL of PEI solution (5 mgmL^-1^) and sonicated for 2 h. The particles were separated from free PEI in solution using a high powered magnet and extensive washing with deionised water. The resultant particles (500 mg) were re-suspended in 5 mL deionised water. Deionised water (400 mL) was stirred on ice. Chloroauric acid (HAuCl_4_, 4%, 375 μL) was added to the water followed by 0.2 M sodium carbonate (Na_2_CO_3_, 500 μL). The solution was stirred for 5 min before addition of sodium borohydride (NaBH_4_, 0.5 mgmL^-1^, 5 mL). The solution turned a deep red colour and was stirred for another 10 min. Fe_3_O_4_-PEI (2 mL, 20 mg) was added to the gold nano-seed solution (90 mL) previously prepared. The solution was stirred at room temperature for 2 h. The magnetic particles were separated from solution and stabilised by stirring in a solution of 0.1 mgmL^-1^ PEI (MW 2000) for 10 min. Finally, the particles (20 mg) were washed extensively with water before re-suspending in 2 mL deionised water. Gold was reduced onto the particle surface forming a complete shell. A solution of sodium hydroxide (NaOH, 0.01 M, 110 mL) was stirred with the particle solution. To this 0.5 mL of 1% HAuCl_4_ was added followed by hydroxyl amine (NH_2_OH·HCl, 0.75 mL, 0.2 M). Four consecutive iterative reductions were carried out by addition of 1% HAuCl_4_ (0.5 mL) and 0.2 M NH_2_OH·HCl (0.25 mL) with 10 min intervals. The final solution was left stirring for 0.5 h before washing in deionised water (x5) and magnetic separation from solution. The particles were resuspended in 10 mL deionised water. A solution of Fe_3_O_4_-PEI-Au (2 mL) was stirred with *O-*[2-(3- Mercaptopropionyl amino) ethyl]-*O*′-methylpolyethylene glycol (PEG-Thiol, 1 mgmL^-1^) for 1 h at 60 °C. The HNPs were washed with deionised water and separated from solution using a high powered magnet. The resultant particles (5 mg) were resuspended in 5 mL deionised water.

### Characterization of HNPs

The iron and gold content of the samples was determined using the inductively coupled plasma (ICP). An acid digestion was carried out on the samples using concentrated nitric acid with heating up to 100 °C (1:1 sample:acid). A calibration was carried out using iron standard and gold standard solutions 0.5 – 5 mgmL^-1^ (R = 0.9999). The samples were diluted with deionized water prior to analysis. A control sample of deionised water was also run. Nanoparticle solutions in distilled water were placed in an ultrasonic bath for 10 min before analysis. Hydrodynamic diameters and polydispersity index measurements were carried out using a photon correlation spectrometer (PCS, Zetasizer Nano-ZS, Malvern Instruments, UK). All measurements were conducted in triplicate at 25 °C and an average value was determined. The zeta potential of the nanoparticles solutions was then analysed to determine their surface charge using the same instrument. Samples diluted in deionised water were dropped onto copper grids (2 μL) and allowed to dry at room temperature. The grids were loaded into the TEM and directly imaged using a JEOL 1200 EX- FDL5000 microscope (Jeol, Japan). High resolution images were captured on a high resolution TEM (HR-TEM), Jeol JEM 2010 (Jeol, Japan). EDX analysis was collected from the HR-TEM images using Link ISIS software. Polymer coated HNP solutions (5 mL) were freeze dried. The resulting powder was run on the FTIR using a diamond tipped attenuated total reflectance attachment (Nicolette iS5 with iD5 ATR, Thermo-Fisher UK). A background scan was run with no sample present. The samples were scanned 64 times and the average spectra recorded. Peak absorbance of samples was determined using a Tecan microplate reader with integrated cuvette port (Infinite M200, Tecan). Aqueous samples were analysed in quartz cuvettes, absorbance scans were carried out between 300 – 700 nm.

### Biological testing of HNPs

A375M human melanoma cells were cultured in RPMI media supplemented with 10% foetal bovine serum (FBS) and 1% penicillin streptomycin (Penstrep) (Invitrogen, UK). The cell viability after incubation with the nanoparticles was determined via direct counting of the viable cells. Cells were grown in 6 well plates and incubated with the magnetic nanoparticles for 24, 72, 120 and 168 h periods. After this time the cells were washed with phosphate buffered saline (PBS) and detached from the well using trypsin. The cells were suspended in fresh media. Trypan blue was added to an equal volume of cell suspension and mixed. 10 μL of trypan blue – cell mixture was pipette into the counting chamber and placed in the automated cell counter (Countess^TM^, Invitrogen, UK). The number of viable cells was recorded and expressed as a percentage compared to total cells (100%).

Cellular uptake of nanoparticles was observed using silver enhanced staining. Briefly, A375M cells were seeded into 6 well plates containing glass coverslips (150,000 cells/well) and grown until 50-60% confluence. Coated magnetic nanoparticles were added to the wells so that the final concentration was 50 ugmL^-1^. The cells were incubated for 24 h, the media was removed and cells were washed with PBS 3 times. Cells were fixed with icy methanol for 10 min followed by treatment of 2.5% HCl (10 min). Cells were washed and treated with LI silver enhanced staining reagents (Invitrogen, UK) for 30 min. Cells were then washed with PBS and coverslips mounted on glass slides. The samples were imaged on a Olympus IX71 light microscope (Olympus, UK) using X40 magnification and Hamamatsu Orca-05 G camera attachment (Hamamatsu, Japan).

### Laser irradiation of HNPs in agar gel

HNPs were evenly dispersed in 2% agar at concentrations of 0.4, 2, 10 and 50 μgmL^-1^. The gels were formed in shallow, 35 mm diameter plastic petri dishes. For exposure, the gel phantom samples at room temperature (22 °C) were exposed to 532 nm continuous wave laser beam emitted by a solid state laser system (Laser Quantum, UK). The 1.1 Watt laser beam was collimated at 7 mm diameter and passed through the centre of the gel, hence the power density of laser irradiation on the sample was ~2.86 Wcm^-2^. The real time temperature change in the gel was monitored by a pair of thermocouples (0.076 mm diameter, T-type, PFA coated, Omega, UK). One thermocouple was positioned at the centre of the gel (in the laser beam) and the second at the edge. A thermocouple logger (TC08 Pico Technology, UK) converted the voltage difference to the real-time change in temperature. The gel samples were positioned in the laser beam using an x,y,z translation stage (Newport, USA) and irradiated by the beam for fixed durations of 20, 40, 90 seconds which were timed using a stopwatch. A control sample of 2% agar was used to measure the temperature change when no nanoparticles were present. The temperature change in the samples was determined as:

(1) $$\Delta \text{ T}=\left({\text{T}}_{\text{final}}–{\text{ T}}_{\text{initial}}\right)-{\text{ T}}_{\Delta \text{ control}}$$

### Magnetic resonance imaging of HNPs

HNPs (0.05 -100 μgmL^-1^) were evenly dispersed 2% agar and placed into 7 mL bijou vials (SLS, UK). The relaxivity measurements were carried out at 19 °C in a 1.5 T clinical MRI scanner (Signa HDx, GE, USA) using GE’s receive-only 8-channel head coil. T_1_ and T_2_ relaxation times were determined using the Inversion Recovery Spin-Echo (IRSE) and Spin-Echo (SE) sequences, respectively. The imaging parameters were as follows IRSE: repetition time (TR) = 15 s; echo time (TE) = 10 ms; acquisition matrix =128x128; field of view (FOV) = 14 cm; band width (BW) = 15.63 kHz; number of excitations (NEX) 1; slice thickness = 10 mm and inversion time (TI) of 100, 200, 400, 600, 800,1000, 1200, 1400 1600, 1800, 2000, and 2200 ms. The SE parameters were as above with TE of 10, 20, 30, 50, 100, 150, 350, 500,750, and 1000 ms. Regions of interest (ROI) were selected in each vial over the image set and Ti (i = 1 and 2) values were calculated from the three-parameter non-linear least squares fit of the mean signal intensities *vs.* time (TI and TE respectively) data. The associated relaxivities (r_i_ in mM^-1^ s^-1^) were obtained from the gradient of the linear least-squares fit of the relaxation rates (R_i_ = 1/T_i_) versus concentration of Fe (mM).

## Competing interests

The authors declare that they have no competing interests.

## Authors contributions

CH carried out core synthesis, characterization of HNPs, parts of cellular assay and laser experiments, supervision of YM and wrote the manuscript. YM carried gold coating and pegylation, cellular assays, sample preparation for laser and MRI experiments, part of laser experiments. MG carried out MRI analysis. CM and AV carried out the laser experiments and AV also involved partially in MRI analysis. PP and ZW were involved in the design and advice of laser treatments, temperature monitoring and hyperthermal application. LW supervised the work and corrected the manuscript. AM and AC were scientific advisors and provided essential facilities and advice, and edited the manuscript. All authors read and approved the final manuscript.
